# Chorioallantoic urothelial tumor avatar. A clinical tool for phenotype-based therapy ^1^

**DOI:** 10.1590/s0102-865020190120000007

**Published:** 2020-02-10

**Authors:** Marina Vian Ossick, Karen Linares Ferrari, Igor Nunes-Silva, Fernandes Denardi, Leonardo Oliveira Reis

**Affiliations:** I MSc, UroScience, Department of Urology, School of Medical Sciences, Universidade de Campinas (UNICAMP), Brazil. Acquisition and analysis of data, manuscript writing.; II MSc, PhD, UroScience, Department of Urology, School of Medical Sciences, UNICAMP, Campinas-SP, Brazil. Acquisition and analysis of data, manuscript writing.; III MD, MSc, Department of Urology, Instituto do Câncer Arnaldo Vieira de Carvalho (IAVC), Sao Paulo-SP, Brazil. Manuscript writing.; IV MD, PhD, UroScience, Department of Urology, School of Medical Sciences, UNICAMP, Campinas-SP, Brazil. Manuscript writing.; V MD, MSc, PhD, UroScience, Department of Urology, School of Medical Sciences, UNICAMP, and; Pontifícia Universidade Católica de Campinas (PUC-Campinas), Brazil. Design of the study, acquisition and analysis of data, manuscript writing.

**Keywords:** Chorioallantoic Membrane, Urinary Bladder Neoplasms, Heterografts, Drug Therapy

## Abstract

In the muscle invasive bladder cancer (MIBC) standard of care treatment only patients presenting a major pathological tumor response are more likely to show the established modest 5% absolute survival benefit at 5 years after cisplatin-based neoadjuvant chemotherapy (NAC). To overcome the drawbacks of a blind NAC (i.e. late cystectomy with unnecessary NAC adverse events) with potential to survival improvements, preclinical models of urothelial carcinoma have arisen in this generation as a way to pre-determine drug resistance even before therapy is targeted. The implantation of tumor specimens in the chorioallantoic membrane (MCA) of the chicken embryo results in a high-efficiency graft, thus allowing large-scale studies of patient-derived “tumor avatar”. This article discusses a novel approach that exploits cancer multidrug resistance to provide personalized phenotype-based therapy utilizing the MIBC NAC dilemma.

## Urothelial cancer and treatments

Bladder cancer corresponds to over 90% of all urothelial tumors and positions 7th and 17th in the tumors ranking, respectively, between men and women. It affects approximately 110.000 men and 70.000 women each year in the world. In Brazil, there were 9.670 new cases in 2016 (7.200 in men and 2.470 in women). In the United States, the American Cancer Association estimates 79.030 new bladder cancer cases diagnosed in 2017^1 , 2^ .

Most urinary bladder carcinomas (BC) are sporadic and arise from the urothelium, which is characterized by a transitional epithelium composed of a layer of superficial (or umbrella) cells, intermediate and basal cells.

More than 70% of BCs present with papillary and non muscle-invasive appearance (NMIBC), including tumors confined to the urothelium (Ta), tumors invading the lamina propria (T1), a thin layer of connective tissue located below the urothelium and carcinoma in situ (pTis or flat hyperplasia); the remaining are muscle-invasive (MIBC)^3 - 5^ .

Cancer treatments can be divided into surgery, radiation therapy, chemotherapy, and immunological treatment, and the choice will depend on the degree of disease progression. In this scenario, bladder cancer treatment strategies are primarily according to TNM disease staging defined after TURB pathological findings. It is already well established that patients diagnosed as pT2 or higher (MIBC) on TURB benefit from neoadjuvant chemotherapy (NAC) before undergoing radical cystectomy (RC). NAC has shown potential to provide an established 5% to 6% absolute survival benefit at 5 years^6 , 7^ .

However, this potential 6% survival benefit seems not to be extendable to all patients unrestrictedly. Only patients presenting a major pathological tumor response after NAC (around 40%) are those who are more likely to show improvements on their overall survival (OS) outcomes^8^ . The remaining 60% of patients who do not present major response to NAC (chemotherapy resistance) are then probably harmed by the treatment due to exposition to NAC side effects and delay to undergo to definitive RC. In addition, up to 40% of patients with MIBC are ineligible for NAC due to renal insufficiency and inadequate performance status secondary to disease evolution^9^ . Therefore, these findings have turned the cisplatin-based NAC efficacy uncertain in the bladder cancer treatment setting and have caused its underutilization worldwide.

In this sense, it still remains of paramount importance to develop novel strategies for MIBC treatment. The development of a precision therapy based on the “precision oncology concept” for MIBC improving NAC effectiveness for patients implies in providing the right treatment to the right patient at the right time, optimizing survival outcomes in a personalized treatment context. Current researches have developed molecular and genetic analyses of tumor tissue aiming to predict response to targeted therapy in order to overcome chemotherapy resistance^10 - 12^ .

In our study, we aimed to develop a preclinical model of urothelial carcinoma in which different sorts of drugs could be pre-tested before clinical application. Patient-derived tumor tissue collected after TURB is grown on a xenograft environment (“tumor avatar”) as a way to pre-determine drug resistance like a chemogram test even before NAC is targeted. This platform approach provides personalized phenotype-based therapy to patients. It has potential to overcome the drawbacks of a blind cisplatin-based NAC possibly leading to improvements on survival outcomes.

## Uroculture test platform

In 1874, Willian Roberts and John Tyndall observed the growth of bacteria in the liquid medium and reported the inhibitory effects of penicillin, thus observing inhibition of the growth of colonies on the agar plate. In 1940, Heatley suggested the use of filter paper discs that contained antimicrobial solutions and Mohs introduced a “radial disc method”. It was the first proposal of evaluation of phenotype of the response of microorganisms to antibiotic treatment, comparing the radius of the proliferation of colonies. After several studies and findings, the World Health Organization (WHO) released a report on the methodology in 1975 and this method is the standard of the International Committee of Clinical Laboratory Standards^13^ .

Currently, the rationale routinely employed in “uroculture” tests can be proposed as a model of phenotypic evaluation of tumor sensitivity to potential personalized treatments filling the gaps between tumor genomic and sensitivity phenotype.

## Chorioallantoic membrane

The chorioallantoic membrane (CAM) assay has been used as a rapid low cost reproducible method to test potential antitumor drugs *in vivo* . This assay has been widely used to study angiogenesis and has also been successfully developed in a tumor xenograft model, including tumors of the pancreas, melanoma, and osteosarcoma, due to the total non-development of the lymphoid system of the embryo, limiting tissue rejection, opening opportunities for new techniques and protocols.

**Figure 1:**
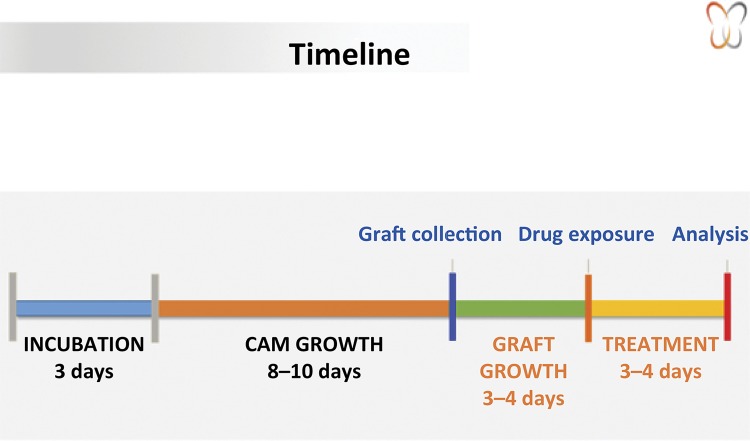
Chorioallantoic tumor avatar timeline.

One of the major challenges for the xenograft model in CAM is the relatively high incidence of embryonic death after egg manipulation, with the mortality rate being between 25 and 50%^14^ . The chicken eggs have been used because they present a favorable environment of vascularization. After the CAM growth, which occurs after 7 days of egg fertilization, access to the blood vessels is highly facilitated, causing them to be manipulated and observed. Figure 1 illustrates a timeline protocol.

This makes possible to sustain tissues and cells of another species and conduct cancer evolution studies. Blood vessels in addition to nourishing the development of allo and xenografts, provide a supportive environment unique to intravasation and dissemination of tumor cells^15^ . The CAM is highly vascularized, which makes it a rich medium to the tumor implant, besides containing other essential proteins and growth factors. This support is unique for invasion, dissemination and vascular arrest of tumor cells^15 , 16^ .

Many of the studies have focused on the development of tumors and the creation of new drugs. The treatment of urothelial carcinoma is still not individualized due to, among many factors, the high tumor heterogeneity. The choice of treatment/drug that the patient will receive takes into account population studies that present inadequacies when transferred to the context of the individualized treatment.

The most commonly used drugs in urothelial carcinoma are MVAC (methotrexate, vinblastine, doxorubicin, and cisplatin) or GC (gemcitabine and cisplatin)^5 , 6^ , simple to apply with low cost and reasonable results. However, when the method is applied to the study of tumors, the results do not represent the favorable conditions of the tumor environment, obliging the cells to an adaptation that interferes in their pattern of growth and response to eventual treatments, so the xenograft models overcome some limitations of the culture, since they provide a solid framework for their development. In contrast, the creation of a xenograft protocol becomes more complex, but it is a more representative method of cancer in its natural environment.

**Figure 2:**
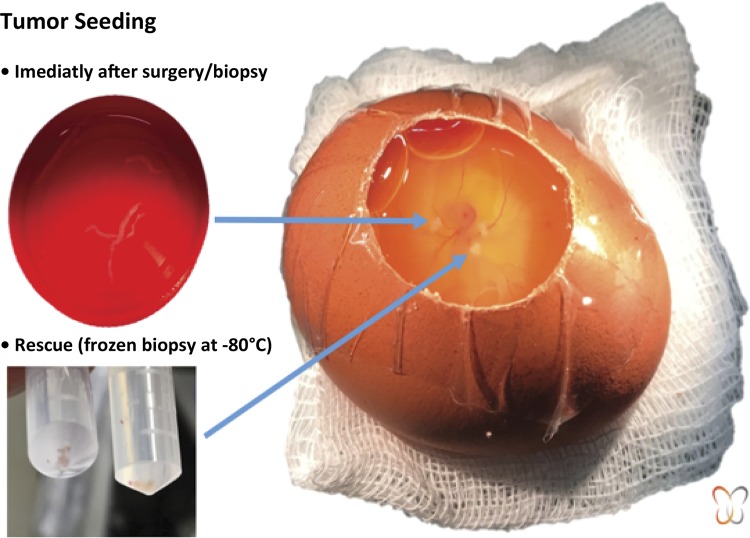
*In-ovo* tumor seeding.

**Figure 3:**
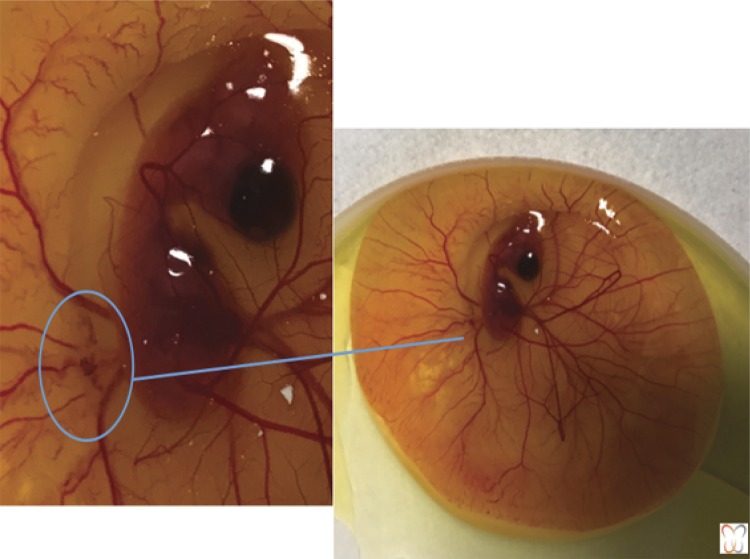
*Ex-ovo* growing urothelial tumor avatar.

In the experimental studies, CAM was studied *in ovo* and *ex ovo* . For *in ovo* studies, the protocol was exposed through a cut of the eggshell^14 , 16 - 20^ , illustrated in Figure 2 . The *ex-ovo* studies address the improvement in CAM and embryo accessibility, allowing its documentation and manipulation^21 , 22^ , illustrated in Figure 3 . This allows other aspects to be monitored, studied and improved, especially regarding angiogenesis and tumor evolution.

## Cancer treatment studies using Patient-Derived Xenograft (PDX)

Seeing the need for more individualized treatment, we fall within the scope of the tumor phenotype, since the same type of tumor can become resistant to different types of drugs, once it has potential to adapt to its host defense mechanisms and characteristics.

Studies adopting xenografts are being used to predict response to certain drugs by using solid tumors from patients implanted in rats and established associations between genotype and drug response in addition to mechanisms of resistance^23 - 25^ .

There is increasing evidence that the heterogeneity of patients and tumors represents the major challenge in the treatment of cancer. Tumor heterogeneity has implications for tumor evolution with functional consequences in terms of drug resistance.

The proposed strategy has the potential to offer patients an ultra-fast response-to-treatment phenotype at different moments of treatment using the chorioallantoic tumor avatar (CTA) platform, with the potential to understand and target the tumor evolution during the treatment history.

As in the context of urinary tract infection where we currently perform a fundamental test of the phenotype of the bacterial response pattern to the treatment (“antibiogram”), we can do the same with tumors, impacting the individualized treatment of patients with potential optimization of oncological, functional and quality of life results with cost reduction, limiting financial toxicity.

## Clinical potential impact

Like the established antibiogram as the main tool in the treatment of urinary tract infection that anticipates the bacteria antibiotic sensitivity/susceptibility, can we define and anticipate the best treatment (”tumor chemogram”) for every individual bladder cancer patient using an ultra-fast treatment response phenotype platform such as chorioallantoic tumor avatar?

**Figure 4:**
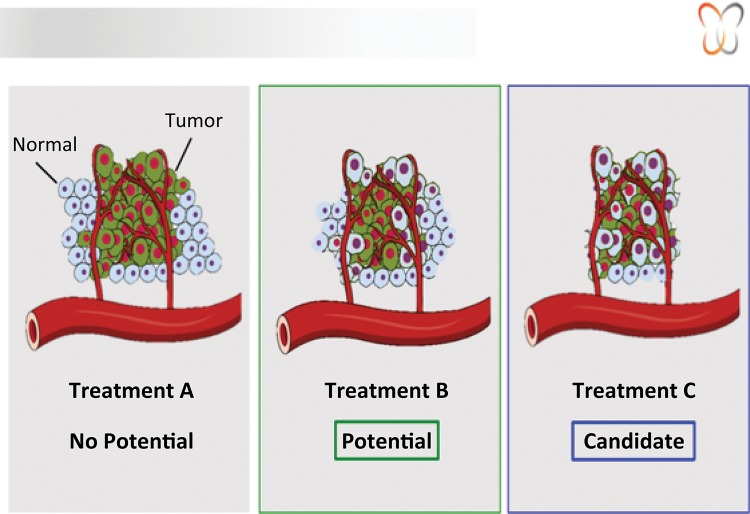
Drug-response phenotype and treatment selection.

Animal model based experience added to clinical practice will further develop the CTA platform to bring it to day by day reality to positively impact patients treatment in the cancer scenario. Figure 4 illustrates the test reading.

**Figure 5:**
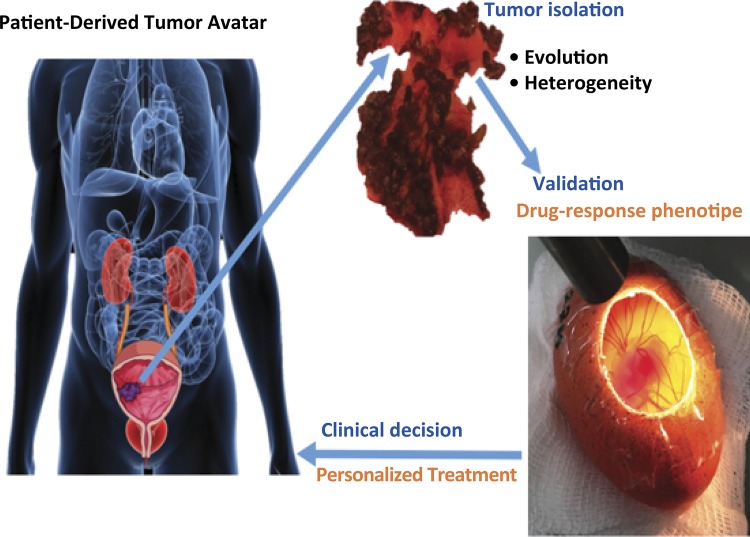
Patient-derived tumor avatar personalized treatment.

The process can be organized in three main steps ( Fig. 5 ):

- Tumor isolation (biopsy, surgery);
- Validation (tumor avatar);
- Clinical decision (personalized treatment).

CTA has the potential to define and anticipate the best treatment (”tumor chemogram”) by a reproducible, relatively low cost and ultra-fast treatment response phenotype platform with impact on clinical practice/disease control allowing a more personalized approach based on patient treatment response phenotype.

## Future challenges and perspectives

The efficiency optimization of the immediate and rescue sowing (in 24 and 48h using frozen tissue -80^o^C) as strategy in cases of failure of the primary sowing, the further understanding of the neoplastic tissue stability and characteristics maintenance (genomic, proteomic, metabolomic and phenotypic) in the proposed platform as well as the definition of this approach impact in the implementation of individualized treatment, its costs and results (oncological and functional) in patients with urothelial carcinoma are underway.

## Footnotes

1 Research performed at Department of Urology, School of Medical Sciences, Universidade de Campinas (UNICAMP), Brazil.
